# Antiproliferative and Anti-Inflammatory Effects of the Polyphenols Phloretin and Balsacone C in a Coculture of T Cells and Psoriatic Keratinocytes

**DOI:** 10.3390/ijms25115639

**Published:** 2024-05-22

**Authors:** Yasmine Ruel, Fatma Moawad, Jérôme Alsarraf, André Pichette, Jean Legault, Davide Brambilla, Roxane Pouliot

**Affiliations:** 1 Centre de Recherche en Organogénèse Expérimentale de l’Université Laval/LOEX, Axe Médecine Régénératrice, Centre de Recherche du CHU de Québec-Université Laval, 1401 18e Rue, Quebec City, QC G1J 2Z4, Canada; yasmine.ruel.1@ulaval.ca; 2 Faculté de Pharmacie, Université Laval, 1050 avenue de la Médecine, Quebec City, QC G1V 0A6, Canada; 3 Faculté de pharmacie, Université de Montréal, 2940, chemin de la Polytechnique, Montreal, QC H3C 3J7, Canada; fatma.moawad@umontreal.ca (F.M.); davide.brambilla@umontreal.ca (D.B.); 4 Laboratoire d’Analyse et de Séparation des Essences Végétales (LASEVE), Centre de Recherche sur la boréalie (CREB), Département des Sciences Fondamentales, Université du Québec à Chicoutimi, 555 boulevard de l’Université, Chicoutimi, QC G7H 2B1, Canada; jalsarra@uqac.ca (J.A.); andre_pichette@uqac.ca (A.P.); jean_legault@uqac.ca (J.L.)

**Keywords:** psoriasis, polyphenols, phloretin, balsacone, anti-inflammatory activity, antiproliferative activity

## Abstract

Plaque psoriasis is a chronic inflammatory skin disease causing red inflamed lesions covered by scales. Leukocytes, including dendritic cells and T cells, participate in the inflammation of the skin by producing multiple cytokines, thus contributing to the hyperproliferation of keratinocytes. Lack of effectiveness and toxic side effects are the main concerns with conventional treatments, and research involving new antipsoriatic molecules is essential. In this study, the anti-inflammatory and antiproliferative effects of two natural polyphenols, phloretin and balsacone C, were investigated using the coculture of T cells and psoriatic keratinocytes. Phloretin exerted antiproliferative activity by regulating the expression of antigen Ki67 and proliferating cell nuclear antigen (PCNA). These effects were comparable to those of methotrexate, a reference treatment for moderate to severe psoriasis. With balsacone C, the expression of Ki67 was also reduced. Additionally, phloretin decreased the levels of multiple pro-inflammatory cytokines: monocyte chemoattractant protein-1 (MCP-1/CCL2), macrophage inflammatory protein-1α (MIP-1α), granulocyte colony-stimulating factor (G-CSF), granulocyte-macrophage colony-stimulating factor (GM-CSF), interleukin-1 alpha (IL-1α), interleukin-1 beta (IL-1β), interleukin-6 (IL-6), interleukin-17A (IL-17A), and tumor necrosis factor alpha (TNF-α). The increased interleukin-2 (IL-2) levels with phloretin and methotrexate also represented anti-inflammatory activity. Balsacone C and methotrexate decreased the levels of IL-1α and IL-1β, but methotrexate exerted a higher reduction. In summary, the anti-inflammatory effects of phloretin were more pronounced than those of methotrexate and balsacone C. In addition, the expression of lymphocyte common antigen (CD45) was more similar to that of the healthy condition after using phloretin or methotrexate. Finally, phloretin stood out from the other compounds and appears promising for psoriasis treatment.

## 1. Introduction

Psoriasis is a chronic inflammatory skin disease [1] characterized by red inflamed lesions with scales [2]. Different regions of the body can be affected including the neck, trunk, arms, legs, head, face, genitals, knees, and elbows, as well as the palmoplantar regions [3,4]. The quality of life of patients can be drastically decreased by the painful lesions, reduced self-confidence, social discomfort, anxiety, and depression linked with their condition [5,6]. Indeed, psychological disorders are not the only comorbidities that patients with psoriasis can suffer. Other inflammatory diseases such as psoriatic arthritis, Crohn’s disease, and uveitis can be associated with psoriasis as well [7]. Many patients suffering from psoriasis are also diagnosed with cardiovascular diseases, obesity, insulin resistance, or dyslipidemia [8]. The prevalence of psoriasis varies from 0.14% in East Asia to 1.99% in Australasia. In other populations from western Europe, central Europe, North America, and southern Latin America, the prevalence was found to be 1.92%, 1.83%, 1.50%, and 1.10%, respectively [9]. In addition, there are many types of psoriasis that affect the skin including plaque, guttate, pustular, inverse, and erythrodermic psoriasis. Other forms of psoriasis can also be manifested including nail psoriasis and psoriatic arthritis [10]. Plaque psoriasis, also named vulgaris psoriasis, is the most common form in about 80–90% of patients [11,12,13]. The psoriatic skin proliferates fast and is much thicker than normal skin [2]; thus, in psoriasis, the epidermis is renewed after 4–5 days [14] compared with 28–56 days for normal skin [15,16,17,18]. The differentiation of the skin is affected, which leads to an inadequate skin barrier [19]. The skin is also infiltered by several leukocytes, especially by T lymphocytes and dendritic cells, and also by neutrophils and macrophages. These immune cells secrete inflammatory molecules such as cytokines, which contribute to the inflammation in the skin, the hyperproliferation of keratinocytes, and, consequently, to epidermal hyperplasia [20].

**Figure 1:**
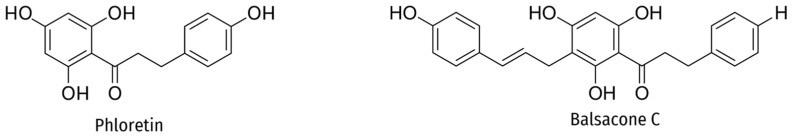
Molecular structure of phloretin and balsacone C.

Although there are several treatments offered to patients suffering from psoriasis (topical creams, systemic treatments, phototherapy, and biological therapies), there is still no cure for it, and patients need to take medication for the rest of their lives. Moreover, the risk of toxicity with the use of long-term treatments is high, and patients can develop multiple side effects. In fact, irritant contact dermatitis can occur with topical treatments, such as the vitamin D analogs tazarotene and dithranol, which is not ideal for skin that is already damaged [21]. Among systemic treatments, the use of methotrexate is well recognized as causing hepatoxicity [22], similar to etanercept as a biological treatment [23]. In addition, the use of biologic treatments, such as adalimumab and infliximab, impacts the immune system and increases the risk of serious infections such as pneumonia, cellulitis, and tuberculosis [24,25]. Also, the risk of developing skin cancers is increased with phototherapy [26]. Furthermore, the efficacy of certain treatments is still not as good as expected, leading to dissatisfaction in 52.3% of patients with psoriasis [27,28]. For instance, 60.2% of patients using methotrexate mentioned its side effects as the top factor attributed to their dissatisfaction [29]. The adverse effects and the lack of effectiveness of biological agents represent the principal reasons for discontinuing these therapies [28]. Thus, considering the unsatisfactory effectiveness and the toxicity of most conventional treatments, research involving new sources of active molecules with antiproliferative, anti-inflammatory, and immunomodulatory properties is relevant for the development of new antipsoriatic products. Recently, the interest in investigating the antipsoriatic proprieties of molecules from natural sources in order to reduce or minimize the side effects has increased. In fact, omega-3 sources, vitamin E, polyphenols, coffee, curcumin, and others have shown anti-inflammatory and antioxidant properties that are of interest for psoriasis treatment [30,31,32]. Indeed, in a previous study with a once-weekly systemic administration of polyphenols named balsacones, a family of dihydrochalcones isolated from the buds of *Populus balsamifera* L., psoriatic reconstructed skin showed improvement and became more comparable to healthy reconstructed skin [33]. With the use of balsacone C, keratinocyte proliferation and skin thickness were reduced, and the differentiation of the skin was improved [33]. In addition, balsacone C showed antibacterial properties [34]. However, the antiproliferative and anti-inflammatory effects of balsacone C have not yet been studied in a psoriatic model with immune cells. Moreover, balsacone C is an analog of phloretin (Figure 1), a polyphenolic molecule abundantly found in fruit (apples, apricots, etc.) [35]. In the literature, phloretin has been reported to show important anti-inflammatory properties. In TNF-α-stimulated HaCaT human keratinocytes, phloretin decreased the production of multiple cytokines including interleukin-6 (IL-6), interleukin-8 (IL-8), regulated on activation, normal T-cell expressed and secreted (RANTES/ CCL5), macrophage-derived chemokine (CCL22/MDC), and thymus and activation-regulated chemokine TARC/ CCL17 [36]. In another study, the oral administration of phloretin in a mouse model of allergic contact dermatitis induced by 2,4-dinitrochlorobenzene (DNCB) allowed the reduction in pro-inflammatory cytokine levels including interleukin-4 (IL-4), IL-6, interleukin-17A (IL-17A), interferon gamma (IFN-γ), and thymic stromal lymphopoietin (TSLP) [37]. Additionally, phloretin was used with HaCaT cells under *Propionibacterium acnes*-induced inflammation and was found to reduce prostaglandin E2 (PGE2) and cyclooxygenase-2 (COX-2) levels [38]. This phenolic compound also had antiproliferative effects on T lymphocytes [39]. However, no study has yet elucidated the anti-inflammatory and antiproliferative potential of phloretin in psoriatic keratinocytes. Thus, the main objective of this study was to determine the anti-inflammatory and antiproliferative effects of phloretin and balsacone C separately in an immunocompetent in vitro psoriatic model of T cells and psoriatic keratinocytes. Methotrexate was chosen as a reference treatment in this study as it is frequently used for moderate to severe psoriasis [40]. To achieve our objectives, cocultures of IL-2-stimulated T cells and psoriatic keratinocytes were chosen as the in vitro psoriatic model based on previous research [41]. This article described inflammatory cytokine levels that were more important in cocultures of T cells and psoriatic keratinocytes with IL-2 supplementation when compared with cocultures without IL-2 supplementation, psoriatic keratinocyte monocultures (with or without IL-2 supplementation), or T cell monocultures (with or without IL-2 supplementation). This study also showed that the interactions between IL-2-stimulated T cells and psoriatic keratinocytes require direct cellular contact [41].

## 2. Results and Discussion

### 2.1. Antiproliferative Potential of Polyphenols and Cellular Metabolic Activity

The sulforhodamine B (SRB) assay was used to determine the antiproliferative potential of the evaluated polyphenols, balsacone C, and phloretin. The experiment was performed with psoriatic lesional keratinocytes from three different human donors with plaque psoriasis (N = three donors, n = six cultures per condition). The percentage of cell growth was calculated for different concentrations of the molecules, and then the median inhibitory concentration (IC_50_) was calculated. The IC_50_ was 125 μM for balsacone C and 166 μM for phloretin (Table 1). This means that with these concentrations, half of the cell growth was inhibited.

A previous experiment using cells from other donors showed that the IC_50_ for balsacone C was 128 μM [33], which is similar to our results. Another study on phloretin measured the IC_50_ using four breast cancer cell lines, and the calculated IC_50_ ranged between 36 and 135 μM, with an average of 91 μM. With four ovarian cancer cell lines, the IC_50_ values ranged between 51 and 197 μM, with an average of 105 μM [42]. There was variability in the donors and the cell lines. To our knowledge, no study has reported an evaluation of the IC_50_ of phloretin with the SRB assay using keratinocytes.

In addition, the level of cellular metabolic activity was calculated using the MTT assay. This experiment was carried out with keratinocytes from three human donors with psoriasis and two healthy human donors (N = 5). The percentage of cellular metabolic activity was calculated as the ratio of the measure obtained with the treatment to that of the control. At the corresponding IC_50_ values of balsacone C and phloretin, the level of cellular metabolic activity was 61% and 73%, respectively (Table 1). The metabolic activity was reduced by both polyphenols in comparison with the control. At the IC_50_, the reduction in cellular proliferation with the SRB assay correlated with the results of the cellular metabolic activity from the MTT test. Indeed, cellular proliferation is associated with metabolic activity [43].

To obtain a sufficient antiproliferative effect in keratinocytes, the median inhibitory concentrations (IC_50_ results from the SRB analyses) were chosen to evaluate the antipsoriatic properties of balsacone C and phloretin in a coculture of psoriatic lesional keratinocytes and T cells and compared with methotrexate. The chosen concentration of methotrexate for the coculture experiment was based on the recommendations of the British Association of Dermatologists’ guidelines for the safe and effective prescribing of methotrexate for skin disease as well as on the recommendations of the National Health Service for patients with psoriasis. The recommended initial dosage is 2.5–15 mg of methotrexate weekly [44,45]. The total dosage used for the coculture was 4 mg weekly, which was delivered as a dose of 1.33 mg on each of the three days of treatment over the period of a week. This is equivalent to a concentration of 734 μM of methotrexate in the culture medium. Previous studies using the same concentration of methotrexate (734 μM) in psoriatic keratinocytes in vitro have reported marked antipsoriatic effects [33,46].

### 2.2. Antiproliferative Effects of Polyphenols on Cocultures of T Cells and Psoriatic Keratinocytes

#### 2.2.1. Ki67 Expression

Antigen Ki67 is a protein expressed during cell cycles, and its expression correlates with proliferation [47]. It can be expressed during the G1 (cell growth), S (DNA synthesis), G2 (more growth, preparation for mitosis), and M (mitosis) phases [48]. Ki67 expression in G0 (resting phase) is absent [49,50]. Ki67 can also be expressed at different levels during mitosis, early G1, mid G1, and late G1 [51]. Ki67 expression is low in G1, but it increases in the S and G2 phases and increases even more during mitosis [48,52]. Additionally, the hyperproliferation of keratinocytes is well characterized in psoriasis [53], where Ki67 is overexpressed compared with normal skin [54]. Moreover, a recent study involving human patients showed that T cells, also named T lymphocytes, can express Ki67 in lesional psoriatic skin in contrast with healthy human skin [55].

**Figure 2:**
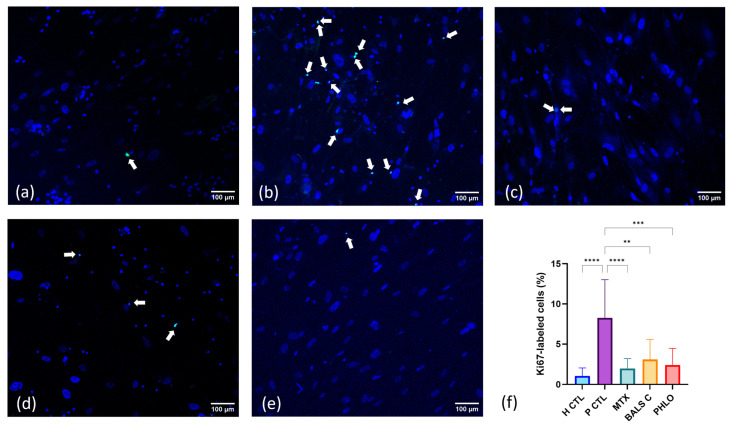
Antiproliferative effects of the polyphenols balsacone C and phloretin compared with those of methotrexate in T cell and psoriatic keratinocyte cocultures. Detection of antigen Ki67 (green) by immunofluorescence staining. White arrows indicate positive cells. Nuclei were stained with DAPI (blue). Bar = 100 μm. The evaluated conditions are (**a**) healthy control, corresponding to healthy keratinocyte monocultures (H CTL), (**b**) psoriatic control, corresponding to a T cell and lesional psoriatic keratinocyte coculture (P CTL), T cell and lesional psoriatic keratinocyte cocultures treated with (**c**) 734 µM of methotrexate (MTX), (**d**) 125 µM of balsacone C (BALS C), and (**e**) 166 µM of phloretin (PHLO). (**f**) The ratios of Ki67-positive cells over the total number of keratinocytes and T cells were calculated (N = 3 donors of healthy keratinocytes or N = 3 donors of psoriatic keratinocytes, n = 2 cocultures per condition, and 3 photos were taken per coculture). Statistical significance was determined using a Kruskal-Wallis test followed by Dunn’s multiple comparisons test. ** *p* < 0.01; *** *p* < 0.001; **** *p* < 0.0001. Each condition is represented in different colors; blue for H CTL, purple for P CTL, followed by the treatments, methotrexate in green, balsacone C in orange and phloretin in red.

To evaluate the antiproliferative effect of balsacone C and phloretin on cocultures of T cells and psoriatic keratinocytes, immunofluorescence staining was performed for Ki67, a proliferation marker (Figure 2). As expected, the expression of Ki67 was statistically higher for the psoriatic control (psoriatic keratinocyte and T cell cocultures, Figure 2b) compared with the healthy control (healthy keratinocyte monocultures, Figure 2a). Our results show that the expression of Ki67 was significantly decreased in psoriatic keratinocyte and T cell cocultures treated with methotrexate (Figure 2c), balsacone C (Figure 2d), and phloretin (Figure 2e) compared with the psoriatic control. This analysis demonstrates that the polyphenols reduce cell proliferation in a 2D psoriatic model.

Phloretin had antiproliferative effects that were close to those of methotrexate, which is known for its antiproliferative activity. Indeed, in a previous clinical study, methotrexate reduced the expression of Ki67 after 1.5 months of treatment [56]. Ki67 expression and the proliferation of keratinocytes are associated with the thickness of the epidermis [57]. A previous report showed a reduction in epidermal thickness when phloretin was administered orally for three weeks in a dermatitis mouse model induced with dinitrochlorobenzene [37]. Further experiments could assess the proliferation of keratinocytes and T cells separately in a psoriatic skin 3D model using balsacone C and phloretin as treatments. In future studies on psoriatic skin, it would be interesting to evaluate if epidermal thickness could be reduced using these polyphenols, thereby resembling that of healthy skin.

#### 2.2.2. PCNA Expression

Proliferating cell nuclear antigen (PCNA) is expressed in the nuclei and participates in DNA synthesis [58]. This proliferation marker is more highly expressed during the G1 and S phases of the cell cycle [59]. On the contrary, quiescent and senescent cells have low PCNA expression [60]. PCNA is also a component of DNA replication and repair mechanisms [59]. In brief, an increased expression of PCNA could signify DNA damage or increased cell proliferation [59]. In addition, the expression of PCNA, as assessed by immunofluorescence staining, in skin from patients with plaque psoriasis is increased compared with healthy controls [61]. The expression of PCNA observed in psoriatic substitutes was also increased compared with their respective controls [62].

**Figure 3:**
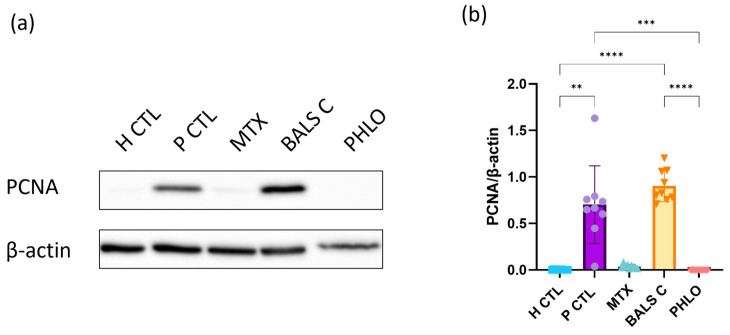
Proliferating cell nuclear antigen (PCNA) expression in the healthy control, corresponding to healthy keratinocyte monocultures (H CTL), psoriatic control, corresponding to a T cell and lesional psoriatic keratinocyte coculture (P CTL), and psoriatic cocultures treated with methotrexate (MTX), balsacone C (BALS C), and phloretin (PHLO). (**a**) For each condition, 10 μg of total protein from the cultures was analyzed by Western blot for the expression of PCNA. β-Actin was used to control equal loading. The analyses were carried out on samples from three different populations of healthy keratinocytes for the healthy control (N = 3) and from three populations of psoriatic keratinocytes for all the psoriatic conditions (N = 3). For each condition, the samples were from three cocultures (n = 3). One representative immunoblot is shown per analyzed protein. (**b**) Densitometric analyses of the immunoblots from panel (**a**) are presented. Statistical significance was determined using a Kruskal-Wallis test followed by Dunn’s multiple comparisons test. ** *p* < 0.01; *** *p* < 0.001; **** *p* < 0.0001. For each condition, different colors and symbols for the data points are represented; blue squares for H CTL, purple circles for P CTL, followed by the treatments, green triangles for methotrexate, orange inverse triangles for balsacone C and red diamond shapes for phloretin.

In the present study, the expression of PCNA was investigated (Figure 3). As expected, the levels of PCNA were higher for the psoriatic control as compared with the healthy control. Phloretin significantly reduced its expression compared with the psoriatic control. Interestingly, phloretin reduced the expression of PCNA to levels comparable to those of the healthy condition, as did methotrexate. This analysis confirms the antiproliferative effects of phloretin in a psoriatic in vitro model with T cells and psoriatic keratinocytes.

To date, some studies have reported Ki67 to be more specific than PCNA for evaluating cell proliferation, as it marks fewer cells [59,63,64]. Briefly, higher levels of PCNA could be associated with DNA synthesis and, because the half-life of PCNA is longer than 20 h, PCNA could still be present in cell nuclei even after its use in the cell cycle [59,65]. Higher levels of PCNA could also be explained by growth factors or DNA damage even if cells are no longer in proliferation [59]. Lastly, the different patterns of Ki67 and PCNA expression in the cell cycle [59] and the prolonged presence of PCNA could be the reasons why balsacone C induced a significant reduction in Ki67 levels and a non-significant change in PCNA levels.

### 2.3. Impact of Polyphenols on Inflammatory Cytokine Secretion

**Figure 4:**
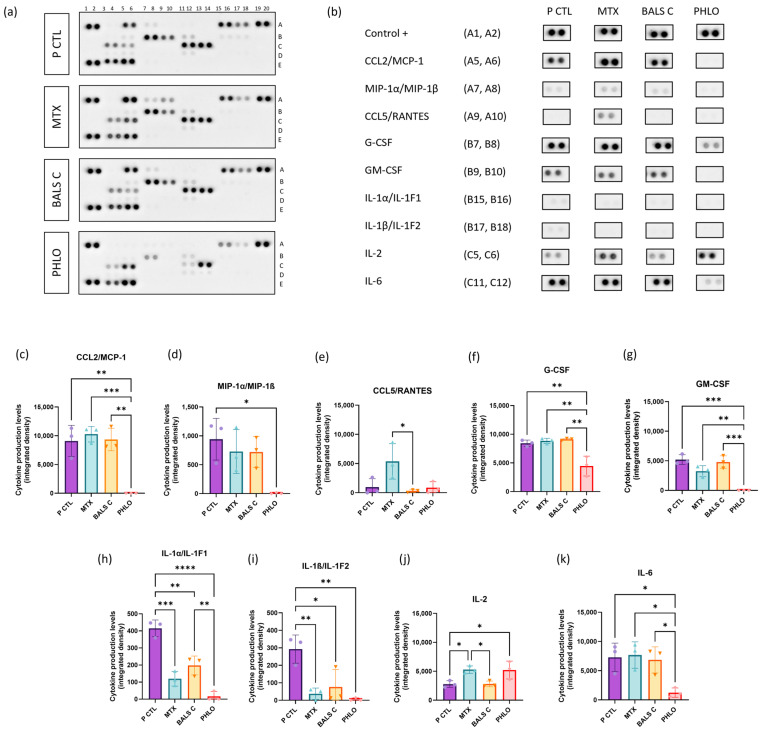
Levels of cytokines in the supernatant of keratinocyte and T cell cocultures. Each treatment was performed on T cell and psoriatic keratinocyte cocultures. Supernatants were collected after the week of treatment was completed. (**a**) Culture supernatants from P CTL, MTX, BALS C, and PHLO underwent analysis for 36 secreted cytokines using the Proteome Profiler Human Cytokine Array kit from R&D Systems™. The darker spots correspond to more expressed cytokines. (**b**) The duplicate spots correspond to the cytokines whose synthesis was the most altered following the treatments. (**c**–**k**) Densitometric analysis of the dot blot duplicates from panel (**b**); (**c**) monocyte chemoattractant protein-1 (MCP-1/ CCL2), (**d**) macrophage inflammatory protein-1 alpha and beta (MIP-1α/MIP-1β), (**e**) regulated on activation, normal T-cell expressed and secreted (RANTES/ CCL5), (**f**) granulocyte-colony-stimulating factor (G-CSF), (**g**) granulocyte-macrophage colony-stimulating factor (GM-CSF), (**h**) interleukin-1 alpha (IL-1α/IL-1F1), (**i**) interleukin-1 beta (IL-1β/IL-1F2), (**j**) interleukin-2 (IL-2), and (**k**) interleukin-6 (IL-6). Data presented are from N = 3 donors of psoriatic keratinocytes per condition. Statistical significance was determined using a Kruskal-Wallis test followed by Dunn’s multiple comparisons test. * *p* < 0.05; ** *p* < 0.01; *** *p* < 0.001; **** *p* < 0.0001. For each condition, different colors and symbols for the data points are represented; purple circles for P CTL, followed by the treatments, green triangles for methotrexate, orange inverse triangles for balsacone C and red diamond shapes for phloretin.

Psoriasis is an inflammatory skin disease with an increased production of multiple cytokines [20]. In this study, the secretion of 36 cytokines was evaluated using the dot blot protocol after the treatment of T cell and psoriatic keratinocyte cocultures (Figure 4a). The cytokines with significant differences between the psoriatic control and at least one of the evaluated treatments are presented with their duplicate spots (Figure 4b) and with their densitometric analyses (Figure 4c–k). One of the cytokines whose levels were the most altered after the treatments was monocyte chemoattractant protein-1 (MCP-1/CCL2). The level of this chemokine was significantly reduced after using phloretin as a treatment on the cocultures compared with the psoriatic control (Figure 4c). This inflammatory molecule is a chemokine produced by keratinocytes, which attracts monocytes and is found at higher levels in the blood of patients with psoriasis [66]. In addition, MCP-1 acts as a chemoattractant for T cells [67]. Phloretin participated in a reduction in MCP-1 production by keratinocytes, thereby reducing the attraction of T lymphocytes. In another study, MCP-1 levels were reduced after adipocytes were treated with phloretin in pro-inflammatory conditions [68]. Other inflammatory molecules that were reduced in quantity using phloretin are macrophage inflammatory protein-1α (MIP-1α) and MIP-1β (Figure 4d). In the literature, it was reported that they are present at higher levels in the blood of patients with psoriasis vulgaris compared with normal blood [69]. MIP-1α and MIP-1β can be secreted by multiple cell types, including macrophages, activated natural killer (NK) cells, and T lymphocytes [70]. MIP-1α levels have also been reduced by using phloretin on adipocytes in pro-inflammatory conditions [68]. CCL5/RANTES is another chemokine involved in the recruitment of T lymphocytes, eosinophils, and monocytes and is produced by keratinocytes as well [71]. RANTES is expressed more abundantly in lesional psoriatic skin than in non-lesional; however, it is not the principal actor in the pathology [72]. In the cytokine array analyses, there were no significant differences in its expression between the psoriatic control and the treatments, including the phenolic compounds and methotrexate. However, the secretion of RANTES was significantly increased with methotrexate compared with balsacone C; either balsacone C had little effect on the secretion of RANTES or methotrexate increased the production of RANTES (Figure 4e). A clinical study in patients with rheumatoid arthritis showed that a six-month administration of methotrexate could increase serum RANTES levels in 40% of the patients [73].

Furthermore, granulocyte colony-stimulating factor (G-CSF) attracts neutrophils and stimulates both their production and activation [74]. This factor is overexpressed in the serum of patients with generalized pustular psoriasis [75], a severe type of psoriasis that leads to the formation of multiple pustules on the epidermis, which are populated by neutrophils [76]. In phloretin-treated cocultures, there was a reduction in G-CSF levels compared with the psoriatic control (Figure 4f). The anti-inflammatory effect of phloretin, as evaluated by G-CSF levels, was also significantly higher than that of methotrexate and balsacone C. A study in which phloretin was administered to peripheral blood mononuclear cells with LPS-induced inflammation also showed a significant reduction in G-CSF levels [77]. In addition, the level of another stimulating factor was significantly reduced with phloretin: granulocyte-macrophage colony-stimulating factor (GM-CSF, Figure 4g). GM-CSF can be expressed by activated T cells, macrophages, and other cell types such as endothelial cells, fibroblasts, and keratinocytes [78,79], and it leads to a pro-inflammatory response [80,81]. GM-CSF is overexpressed in the plasma of patients with moderate to severe psoriasis compared with healthy subjects [82]. This molecule is also overexpressed in the skin of patients with vulgaris psoriasis compared with healthy skin, as well as in those with erythrodermic psoriasis, pustular psoriasis, and palmoplantar pustulosis [79]. Interestingly, phloretin disrupted the production of GM-CSF. Conversely, methotrexate insignificantly reduced the expression of GM-CSF. In another study, in cultures of peripheral blood mononuclear cells with LPS-induced inflammation and supplemented with phloretin, GM-CSF levels were lower but not significantly different [77].

In addition, in the present study, the interleukin-1 alpha (IL-1α/IL-1F1) was less strongly expressed in the psoriatic control than the other cytokines described here, but it was associated with changes in secretion levels for every treatment. Balsacone C reduced IL-1α/IL-1F1 secretion, but the reference treatment, methotrexate, gave a higher reduction. The most significant decrease in IL-1α levels was with phloretin (Figure 4h). Surprisingly, this cytokine is less strongly expressed in lesional psoriatic skin than in non-lesional and healthy skin, contrary to the β form [83]. Clinical studies showed that the levels of this cytokine can also be reduced in lesional psoriatic skin by using systemic retinoids [83]. On the other hand, when using a combination of systemic methotrexate and folic acid, the levels increased to IL-1α levels after treatment that were more similar to those of non-lesional skin [84]. In our study, each treatment reduced IL-1α levels, and interestingly, the graphical appearance of IL-1α production looked like the one for GM-CSF. IL-1α can be secreted by keratinocytes and synergizes with GM-CSF to maintain the stimulation of Langerhans cells and their interaction with T cells [85,86]. IL-1α can also stimulate the differentiation of naive CD4+ T lymphocytes into T helper 17 (Th17) cells [85]. Another cytokine that takes part in the activation of T cells is the interleukin-1 beta (IL-1β/IL-1F2). It stimulates the production of IL-17A by these cells. IL-1β also interacts with keratinocytes, which leads to the secretion of chemokines. Also, IL-1β is very abundant in psoriatic skin compared with healthy skin [87]. According to the cytokine array assays, all the treatments significantly reduced the secretion of IL-1β, but the levels were significantly lower with an administration of phloretin or methotrexate than with balsacone C (Figure 4i). A clinical study with systemic methotrexate showed a reduction in IL-1 β levels in the lesional skin of patients with psoriasis after 12 weeks of treatment to levels more comparable to those in non-lesional skin [84]. In peripheral blood mononuclear cells with LPS-induced inflammation, there was no significant reduction in IL-1β levels after the administration of phloretin, contrary to the results for IL-1α [77]. In our study, IL-1β expression was reduced using phloretin. This could be explained by the presence of T cells in our cocultures and by the reduction in other T cell chemoattractant levels, as described above for MCP-1.

Methotrexate and phloretin led to a higher expression of interleukin-2 (IL-2), but its expression was not affected by balsacone C compared with the psoriatic control (Figure 4j). It is important to mention that supplementation of 30 U/mL of recombinant human IL-2 (rhIL-2) was added to the culture medium as a growth factor for T cells [88] and to ensure their proliferation, as has already been reported [41,89]. A second advantage of rhIL-2 supplementation for cocultures of T cells and psoriatic keratinocytes is the obtention of a pro-inflammatory profile that is more representative of psoriasis [41]. In another study, the dermal injection of activated immunocytes with 20 U/mL of IL-2 contributed to an increase in the psoriatic phenotype of a severe combined immunodeficient mouse model with human non-lesional skin xenografts. This resulted in a thicker epidermis in comparison with an injection of unstimulated immunocytes, as well as in T cell infiltration in the epidermis [90]. Furthermore, IL-2 can exert pro-inflammatory or anti-inflammatory effects depending on its levels; this is called the IL-2 paradox [88]. It also involves the fundamental role of IL-2 in suppressing immune responses [88]. For example, a dosage of 100 U/mL of rhIL-2 in wild-type C57Bl6 mice inhibited the polarization of Th17 cells in contrast to a dosage of 10 U/mL, and it indirectly constrained IL-17 secretion [91]. Moreover, it has been demonstrated that patients with psoriasis have an imbalance between T reg cells and Th17 cells; T reg cells are less effective, and Th17 cells are produced in greater numbers [92]. Surprisingly, in a clinical study, even if there was no imbalance in IL-2 between healthy volunteers and patients with psoriasis, using an IL-2 dose of 0.5 million U (corresponding approximately to 100 U/mL, assuming 5000 mL of blood in the human body) as treatment resulted in an increased production of T regulatory cells, a decrease in Th17 cell numbers and a reduction in quantities of the pro-inflammatory molecules IL-6, IL-17, IFN-γ, and Tumor Necrosis Factor alpha (TNF-α) [93]. In the present coculture study, the increased production of IL-2 after phloretin or methotrexate treatment could have contributed to reducing the polarization of Th17 cells and increasing the production of T regulatory cells, which could then have contributed to the reduction in inflammation, but this hypothesis will have to be assessed with further experiments. Many studies have also demonstrated that polymorphisms could modify IL-2 signaling in humans and that these polymorphisms were also associated with autoimmune diseases [94,95].

One last cytokine that had significant changes in its levels after treatment of the cocultures was IL-6. Only phloretin reduced the expression of IL-6 (Figure 4k). This cytokine is known to stimulate the proliferation and differentiation of keratinocytes [96,97,98]. This pro-inflammatory molecule is also more highly expressed in lesional psoriatic skin than in non-lesional tissues [96]. Furthermore, there were higher levels of IL-6 in the plasma of patients with psoriasis contrary to no detectable bioactivity in normal volunteers [96]. Another study also confirmed that patients with psoriasis had higher levels of IL-6 in their blood in contrast to healthy volunteers [93]. The injection of 5 mg/kg and 20 mg/kg of phloretin in mice with lipopolysaccharide (LPS)-induced acute lung injury resulted in a reduction in the expression of IL-6 and TNF-α [99], which is consistent with our results. The densitometric analyses of all the 36 cytokines evaluated are presented in the Supplementary Materials (Figure S1).

Finally, the cytokine array analyses from the cocultures of T cells and psoriatic keratinocytes demonstrated that balsacone C significantly decreased the levels of IL-1α and IL-1β compared with the psoriatic control; however, methotrexate led to a higher reduction. With phloretin, the levels of multiple cytokines were significantly reduced including CCL2/MCP-1, MIP-1α/MIP-1β, G-CSF, GM-CSF, IL-1α, IL-1β, and IL-6. Additionally, with phloretin and methotrexate, the levels of an anti-inflammatory cytokine, IL-2, increased. Thus, phloretin exerted anti-inflammatory effects in cocultures of T cells and psoriatic keratinocytes that seemed to be more pronounced than those of methotrexate and balsacone C.

**Figure 5:**
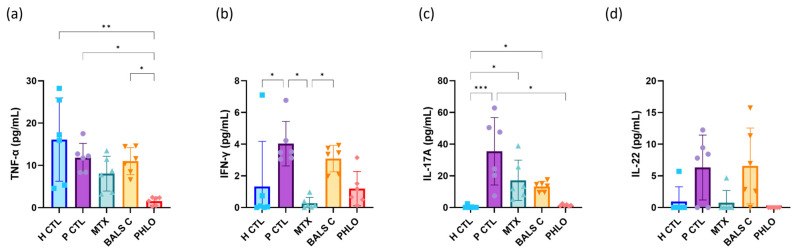
Levels of inflammatory cytokines in the supernatant of healthy keratinocyte monocultures (H CTL) as well as T cell and psoriatic keratinocyte cocultures (P CTL, MTX, BALS C, and PHLO). ELISA analyses were used to detect different secreted cytokines in culture supernatants collected after 7 days of treatment. The impact of the different treatments on keratinocyte and T cell production of (**a**) tumor necrosis factor alpha (TNF-α), (**b**) interferon-gamma (IFN-γ), (**c**) interleukin-17A (IL-17A), and (**d**) interleukin-22 (IL-22) was measured using, respectively, the TNF-α Human ELISA Kit, the IFN-γ Human ELISA Kit, the IL-17A Human ELISA Kit and the IL-22 Human ELISA Kit from Invitrogen™ (ThermoFisher Scientific, Waltham, MA, USA). Data presented are for N = 3 donors of healthy keratinocytes or N = 3 donors of psoriatic keratinocytes. Each condition was evaluated using the supernatant of two cocultures (n = 2 per condition) in duplicate as per the recommendations of the protocol. Statistical significance was determined using a Kruskal-Wallis test followed by Dunn’s multiple comparisons test. * *p* < 0.05; ** *p* < 0.01; *** *p* < 0.001. For each condition, different colors and symbols for the data points are represented; blue squares for H CTL, purple circles for P CTL, followed by the treatments, green triangles for methotrexate, orange inverse triangles for balsacone C and red diamond shapes for phloretin.

To further verify the anti-inflammatory effects of the evaluated treatments, the main cytokines involved in the pathogenesis of psoriasis were directly quantified by ELISA. T cells play an important role in the disease, and they secrete many cytokines that increase the level of inflammation, in particular, TNF-α, IFN-γ, IL-17A, and interleukin-22 (IL-22) [20]. In the coculture experiment, TNF-α secretion was significantly reduced when phloretin was used compared with the psoriatic control (Figure 5a). A previous study also noticed a reduction in TNF-α production after using phloretin on human colonic epithelial cells [100]. Phloretin also reduced the expression of IFN-γ, but in contrast to methotrexate, this was not a significant change (Figure 5b). Methotrexate was reported to reduce IFN-γ expression in patients with rheumatoid arthritis [101]. In the present coculture experiment, IL-17A secretion was significantly reduced with phloretin, and the quantities were comparable to those of the healthy control (Figure 5c). In line with our results, a previous study mentioned that the oral administration of phloretin over three weeks reduced the levels of multiple cytokines, including IL-17A, INF-γ, and IL-6, in mice with dinitrochlorobenzene-induced dermatitis [37]. Furthermore, IL-22 secretion was also reduced by phloretin and methotrexate in our study, and the results for both treatments were comparable to the healthy control (Figure 5d). However, the reduction was not significant for either phloretin or methotrexate when compared to the psoriatic control. Balsacone C did not change IL-22 levels. The levels of the inflammatory cytokines TNF-α, IFN-γ, IL-17A, and IL-22 are presented for every donor separately in the Supplementary Materials (Figure S2 for the healthy donors and Figure S3 for the donors with psoriasis).

In summary, phloretin exerted anti-inflammatory effects by decreasing the expression of the main cytokines involved in the disease in cocultures of T lymphocytes and psoriatic keratinocytes. The levels of TNF-α and IL-17A were significantly reduced compared with the psoriatic control, and IFN-γ and IL-22 levels were lower as well. Methotrexate significantly reduced IFN-γ levels, and IL-22 levels were also lower. According to the ELISA analyses, balsacone C did not significantly change the expression of the evaluated cytokines. In this experiment, phloretin again reduced the expression of several inflammatory molecules, generally to a greater extent than methotrexate and balsacone C. The anti-inflammatory effects of phloretin measured here are significant and consistent with previous reports. For instance, after the administration of phloretin, cultures of peripheral blood mononuclear cells with LPS-induced inflammation showed a significant reduction in the levels of several pro-inflammatory molecules including TNF-α, IL-1α, IFN-γ, CCL2, CCL5, C-X-C motif chemokine ligand 5 (CXCL5), G-CSF, interleukin-10 (IL-10), and interleukin-1 receptor antagonist (IL-1Ra) [77]. Phloretin also reduced the expression of IL-8, TNFα, IL-1β, and IL-6 in cocultures of intestinal epithelial cells and macrophages in LPS-induced inflammatory conditions [102]. Overall, our results suggest that phloretin is an effective anti-inflammatory compound in a psoriatic immune in vitro model. The anti-inflammatory activity of phloretin stood out in general from balsacone C and methotrexate, a reference treatment for moderate to severe psoriasis.

**Figure 6:**
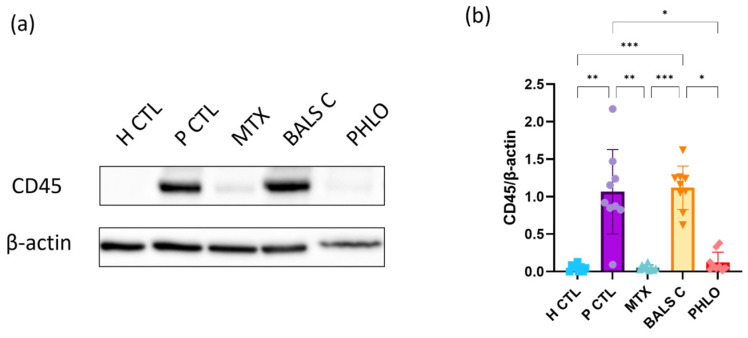
Lymphocyte common antigen (CD45) expression in healthy control, healthy keratinocyte monocultures (H CTL), and T cell and psoriatic keratinocyte cocultures; psoriatic control (P CTL) and cells treated with methotrexate (MTX), balsacone C (BALS C), and phloretin (PHLO). (**a**) For each condition, 10 μg of total protein from the cultures was analyzed by Western blot for the expression of CD45. β-Actin was used as a loading control. The analyses were carried out on samples from three different donors of healthy keratinocytes for the healthy control (N = 3) and from three donors of psoriatic keratinocytes for all the psoriatic conditions (N = 3). For each condition, the samples were from three cocultures (n = 3). One representative immunoblot is shown per analyzed protein. (**b**) Densitometric analyses of the immunoblot from panel (**a**). Statistical significance was determined using a Kruskal-Wallis test followed by Dunn’s multiple comparisons test. * *p* < 0.05; ** *p* < 0.01; *** *p* < 0.001. For each condition, different colors and symbols for the data points are represented; blue squares for H CTL, purple circles for P CTL, followed by the treatments, green triangles for methotrexate, orange inverse triangles for balsacone C and red diamond shapes for phloretin.

In addition, to investigate if the treatments under study could reduce the contact between T lymphocytes and psoriatic keratinocytes in coculture [41], analyses of lymphocyte common antigen (CD45) expression by Western blot were carried out (Figure 6). This protein is expressed on leukocytes, including T cells [103]. It is also known that in psoriatic skin, the infiltration of T cells into the dermis and the epidermis leads to higher levels of CD45 expression in comparison to healthy skin [89]. In the present study, as expected, the psoriatic control contained higher levels of CD45 compared with the healthy control. Following the treatment with phloretin, CD45 levels were significantly reduced, but methotrexate produced an even lower expression of CD45. There were no significant differences between the healthy control and cultures treated with phloretin or methotrexate. However, balsacone C treatment did not change the expression of CD45. Our results suggest that after one week of treatment with phloretin or methotrexate, the presence of T cells was reduced, and this could also signify that the contact between T cells and psoriatic keratinocytes was less important in these cocultures. These findings confirm the anti-inflammatory effects of phloretin and methotrexate in a psoriatic in vitro model of T cells and psoriatic keratinocytes.

## 3. Materials and Methods

### 3.1. Compound Preparation

Balsacone C was prepared as described previously [104,105]. The molecule was extracted from *Populus balsamifera* L. buds, and the molecule was purified to over 95%. Phloretin was obtained from a natural source in purity ≥98% [106]. The compounds were diluted in 0.1% (*v*/*v*) dimethylsulfoxide (DMSO; Sigma, Oakville, ON, Canada) and stored at –20 °C.

### 3.2. Skin Biopsies and Donors

Ethical approvals were obtained in compliance with the Research Ethics Committee of the Centre Hospitalier Universitaire (CHU) de Québec, and the biopsies were performed according to the guidelines of the Declaration of Helsinki. The patients received adequate information, and their signatures were obtained with their consent. The keratinocytes used for the experiments were isolated from the epidermis of human skin biopsies. To isolate the keratinocytes, the skin biopsies were first submerged in thermolysin, which selectively digested the dermo-epidermal junction. Then, an incubation in trypsin broke down the desmosomes of the epidermis to separate the keratinocytes [107].

For the antiproliferative and cell viability assays, the psoriatic keratinocytes used were from psoriatic lesional skin biopsies of donors with chronic plaque psoriasis (psoriasis vulgaris). They were from one male aged 49 years old and females aged 36 and 69 years old (N = 3). The information about the donors with plaque psoriasis is in Table 2. For the cell viability analysis, in addition to psoriatic cells, cells from healthy donors were also used; these donors were females aged 22 and 23 years old who had breast reduction surgery (N = 5).

For the coculture experiment with keratinocytes and T cells, the psoriatic keratinocytes were isolated from psoriatic lesional biopsies of donors with psoriasis vulgaris. The donors were one male aged 46 years old and females aged 65 and 69 years old (N = 3). The information about the donors with plaque psoriasis is in Table 2. The keratinocytes used for the healthy control came from skin biopsies of three healthy donors (N = 3) who had breast reductions. The donors were all females aged 22, 46, and 52 years.

### 3.3. Cell Culture Medium

Culture conditions were inspired by recent works [41,89]. Keratinocytes and T cells were cultured in three parts Dulbecco’s modified Eagle’s medium (DMEM; Thermo Fisher Scientific, Waltham, MA, USA) with one part Ham’s F12 (Thermo Fisher Scientific, Waltham, MA, USA) supplemented with 5% Fetal Clone II serum (HyClone; Thermo Fisher Scientific, Waltham, MA, USA), 100 UI/mL penicillin G (Sigma, Oakville, ON, Canada), 25 µg/mL gentamicin (Schering, Pointe-Claire, QC, Canada), 0.4 µg/mL hydrocortisone (Calbiochem, EMD, Biosciences, Gibbstown, NJ, USA), 5 µg/mL insulin (Sigma, Oakville, ON, Canada), 10 ng/mL human epidermal growth factor (EGF; Austral Biological, San Ramon, CA, USA), and $10^{-10}$ M cholera toxin (MP Biomedicals, Montreal, QC, Canada).

### 3.4. Antiproliferative Potential and Cellular Metabolic Activity

The antiproliferative potential of the polyphenols balsacone C and phloretin on psoriatic keratinocytes was evaluated with the sulforhodamine B (SRB) (Sigma, Oakville, ON, Canada) assay. Psoriatic keratinocytes (N = 3 donors) at passage 2 were seeded on a feeder layer of irradiated human fibroblasts until they reached 85% confluence. Then, the cells were dissociated with trypsin and seeded at 5 × 10 ^3^ cells per well of 0.33 cm² in a 96-well plate on a feeder layer of irradiated human fibroblasts. After 48 h, the keratinocytes were treated with increasing concentrations (6.25, 12.5, 25, 50, 100 μg/mL) of the different compounds (n = 6 wells for each concentration and each molecule). The polyphenols were dissolved separately in DMSO at 100,000 μg/mL (Sigma, Oakville, ON, Canada), and this solution was added to the culture medium. The final concentration of DMSO in the culture medium was maintained at less than 0.1% (*v*/*v*). After 48 h of treatment, keratinocytes were fixed with a 50% solution of trichloroacetic acid (Bio Basic, Markham, ON, Canada), and the plates were placed at 4 °C for two hours. The plates were washed with demineralized water. Then, the cells were dyed with a 0.1% SRB (Sigma, Oakville, ON, Canada) solution. The SpectraMax^®^ Plus 384 Absorbance Plate Reader was used to determine the absorbance of the solution in each well at 540 nm (Molecular Devices, San Jose, CA, USA). The percentage of cell growth was calculated using the method outlined in [108]. The median inhibitory concentration (IC_50_) for balsacone C and phloretin was calculated, and these concentrations were used thereafter in the coculture of T cells and psoriatic keratinocytes experiment.

The cellular metabolic activity of cultures treated with different concentrations of balsacone C and phloretin separately was determined by a 3-(4,5-dimethylthiazolyl-2)-2,5-diphenyltetrazolium bromide (MTT) assay. This analysis was conducted using keratinocytes from three donors with psoriasis and two healthy donors (N = 5). The cells were seeded using the same methodology as the SRB protocol. The treatments were carried out for 48 h. The concentrations evaluated were 6.25, 12.5, 25, 50, and 100 μg/mL (n = 6 wells for each concentration and each molecule). Afterward, the plates were washed three times with 1X phosphate-buffered saline (PBS). An MTT (Sigma, Oakville, Ontario, Canada) solution of 0.5 mg/mL was added to each well, and the plates were incubated at 37 °C, 8% CO_2_ for three hours. Then, the solution was aspirated and a fresh solution of isopropanol and hydrochloric acid (HCl) was added. After 30 min, the crystals were dissolved, and the absorbance was read with a SpectraMax^®^ Plus 384 Absorbance Plate Reader at a wavelength of 570 nm.

### 3.5. Isolation and Activation of T Cells

Blood from a healthy donor was collected according to the guidelines of the Research Ethics Committee of the Centre Hospitalier Universitaire (CHU) de Québec and according to the Declaration of Helsinki. T cells were isolated from the blood and then activated as described previously [89]. The EasySep™ Direct Human T Cell Isolation Kit (StemCell Technologies, Vancouver, BC, Canada) was used to isolate T cells from the blood by immunomagnetic negative selection according to the manufacturer’s instructions. The T cells were then activated using phorbol 12-myristate 13-acetate (PMA) and ionomycin. The T cells were submerged in DMEM culture medium supplemented with PMA (25 ng/mL, Sigma-Aldrich, St-Louis, MO, USA) and ionomycin (1 µg/mL, Sigma-Aldrich, St-Louis, MO, USA) for 4 h at 37 °C, 8% CO_2_.

### 3.6. Coculture of Keratinocytes and T Cells

All cultures were kept in an incubator at 37 °C under 8% CO2. Irradiated human fibroblasts were seeded in 6-well plates (8 × 10 ^4^ cells/well) for one week of culture as a feeder layer for keratinocytes. Keratinocytes (at passage 3) were seeded in these 6-well plates at 1 × 10 ^6^ cells/well. Then, the activated T cells were added at 5 × 10 ^5^ cells/well with 30 U/mL of recombinant human IL-2 (R & D Systems, Minneapolis, MN, USA). The day after, the three different treatments were applied separately as follows: methotrexate, as a reference treatment for moderate to severe psoriasis, and the two polyphenols, balsacone C and phloretin. After two days, there was another treatment application, and after two more days, the last treatment was applied. The total treatment time was one week, and there were three administrations of each treatment for each condition (medium changes at the same time). The culture medium of the control conditions was also changed at the same time. To avoid T cell loss during medium changes, the culture medium was collected in tubes and centrifuged (700× *g*) for 10 min. The medium was aspirated, and a new medium was added to the cell pellets, precipitated in the bottom of the tubes. The T cells were then reseeded in the 6-well plates with keratinocytes. For the treatment with methotrexate (reference compound), the culture medium was supplemented to maintain a final concentration of 734 μM (Injectable USP Methotrexate, 25 mg/mL, Galenova, Saint-Hyacinthe, QC, Canada). The polyphenols were dissolved individually in DMSO (100 mg/mL) and added to their respective culture media. The final concentration of DMSO in the culture medium was maintained at less than 0.05% (*v*/*v*). The concentrations used for the polyphenols were their respective IC_50_ determined with the SRB experiments as follows: balsacone C, 128 μM and phloretin, 163 μM. Additionally, a healthy control represented by healthy keratinocyte monocultures was compared with the cocultures.

### 3.7. Immunofluorescence Analyses

Irradiated human fibroblasts were seeded in 24-well plates (1.6 × 10 ^4^ cells/well) for one week of culture as a feeder layer for keratinocytes. After one week of treatment in the 6-well culture plates, cells from the 6-well culture plates were dissociated with trypsin to detach them from the surface, and they were then seeded in the 24-well cell culture plates at 5.7 × 10 ^4^ cells/well, keeping each condition in different wells. After 24 h of culture at 37 °C and 8% CO_2_, the cells were fixed with 0.5 mL of 4% paraformaldehyde (Electron microscopy sciences, Hatfield, PA, USA) per well for 1 h. The wells were washed three times with 1X PBS. Indirect immunofluorescence staining was performed on the fixed cells in the well plates. The antibodies were prepared using PBS containing 1% bovine serum albumin (BSA). Immunofluorescence staining was performed for Ki67; the primary antibody used was mouse monoclonal anti-Ki67 (IgG1) (dilution 1:400, Biosciences, Mississauga, ON, Canada), and the secondary antibody was goat anti-mouse IgG (H+L)—Alexa Fluor™ 488 (dilution 1:1000, Life Technologies, Eugene, OR, USA). Afterward, the mounting medium DAPI Fluoromount-G (SouthernBiotech, Birmingham, AL, USA) was added to the wells to label cell nuclei. The stained cells were observed with a Zeiss LSM 700 Confocal (Zeiss, Oberkochen, Germany) 10x objective. The ratio of Ki67-positive cells (in green, indicated with white arrows) to the total number of cell nuclei (in blue) was calculated. (Immunofluorescence staining was performed on N = 3 donors of psoriatic keratinocytes or N = 3 donors of healthy keratinocytes, n = 2 cocultures per condition, and 3 photos were taken per coculture.)

### 3.8. Western Blot Analysis

At the end of the week of treatment, the supernatant from the 6-well plates was aspirated, and RIPA buffer containing the cOmplete™ protease inhibitor cocktail (Roche, Diagnostics GmbH, Germany) was added to the cells to extract the total protein. The plates were incubated on ice for 5 min, and afterward, the samples were collected in tubes and centrifuged at 12,000× *g* for 20 min at 4 °C. The supernatants were collected and placed at −80 °C until the next steps were carried out. The samples were concentrated using the Pierce Protein Concentrator PES (3K or 5K MWCO, 0.5–100 mL, ThermoFisher Scientific, Waltham, MA, USA). The dosage of the samples was performed using a Micro BCA Protein Assay Kit (ThermoFisher Scientific).

Western blot analyses were carried out to compare the expression of PCNA (1:2500, ab29, Abcam, Cambridge, MA, USA) and CD45 (1: 500, ab10558, Abcam, Cambridge, MA, USA) in the coculture of T cells and psoriatic keratinocytes with or without treatment in contrast to the culture of healthy keratinocytes. For these analyses, ten percent polyacrylamide gels were prepared, and 10 μg of total protein was loaded into the gels. The PageRuler Prestained Protein Ladder, 10 to 180 kDa (ThermoFisher Scientific) was used. The migration was carried out at 100 Volts for 2 h. The gels were transferred onto Immun-Blot PVDF membranes (Bio-Rad Laboratories, Mississauga, ON, Canada) at 25 Volts and 4 °C overnight. Each membrane was incubated with tris-buffered saline (TBS) containing 5% non-fat milk and 0.05% Tween 20 (Sigma, Oakville, ON, Canada) for 1 h. Then the primary antibody was added for 1 h of incubation for PCNA and overnight for CD45. The membranes were then incubated with the secondary antibody for 1h, either anti-mouse HRP antibody for PCNA (1:30,000, 115-035-003, Jackson Immuno Research Laboratories Inc., West Grove, PA, USA) or anti-rabbit HRP antibody for CD45 (1:30,000, 111-035-003, Jackson Immuno Research Laboratories Inc., West Grove, PA, USA). The detection of the proteins on each membrane was performed using the Amersham ECL Prime Western Blotting Detection Reagent (Cytiva, Marlborough, MA, USA). To view the bands from the membrane blots by chemiluminescence, a Fusion Fx7 imager (MIB Lab Equipment, Kirkland, QC, Canada) was used. The amounts of protein were quantified using Image J software version 2.14.0.

### 3.9. Cytokine Array

The Proteome Profiler Human Cytokine Array Kit (R & D Systems, Minneapolis, MN, USA) was used to quantify 36 cytokines in four different conditions following the manufacturer’s procedure. The cell culture supernatants were collected at the end of the week of treatments and stored at –80 °C until used for the cytokine array (900 μL per evaluated conditions). The detection of the cytokines on the membranes was performed using Fusion FX7 (MIB Lab Equipment, Kirkland, QC, Canada) over a period of 10 min. The quantification of the cytokines by the integrated density of the spots (densitometry) was performed using Image J software version 2.14.0. The conditions evaluated were for three psoriatic donors (N = 3).

### 3.10. ELISA Analyses

The levels of secretion of specific cytokines into the culture medium were determined with ELISA analyses. They were performed on cell culture supernatants collected at the end of the week of treatments. The levels of IFN-γ, TNF-α, IL-17A, and IL-22 were evaluated with the IFN gamma Human ELISA Kit (ThermoFisher Scientific, Waltham, MA, USA), the TNF alpha Human ELISA Kit (ThermoFisher Scientific, Waltham, MA, USA), the IL-17A Human ELISA Kit (ThermoFisher Scientific, Waltham, MA, USA), and the IL-22 Human ELISA Kit (ThermoFisher Scientific, Waltham, MA, USA). For these assays, volumes of 50 μL, 100 μL, 100 μL, and 100 μL of supernatant, respectively, were used for each sample. Psoriatic keratinocytes from three donors (N = 3) or healthy keratinocytes from three donors (N = 3) were used, and for each condition, the supernatants of two cocultures (two different samples) were used (n = 2 per condition). For each condition, six samples were analyzed in duplicate, and an average was calculated for each sample. The spectrophotometer SpectraMax^®^ Plus 384 Absorbance Plate Reader was used to determine the absorbance of the samples at 450 nm (Molecular Devices, San Jose, CA, USA).

### 3.11. Statistical Analysis

Statistical analyses were performed using Prism software 9.5.0. (GraphPad Prism Software, San Diego, CA, USA). The data were expressed as means ± standard deviation. Statistical significance was determined using a Kruskal-Wallis test followed by Dunn’s multiple comparisons test. Values of *p <* 0.05 were considered statistically significant.

## 4. Conclusions

This study identified two polyphenols, phloretin and balsacone C, with antiproliferative and anti-inflammatory properties on the cocultures of T cells and psoriatic keratinocytes. However, phloretin stood out from balsacone C and methotrexate, the reference treatment. Phloretin reduced the expression of the proliferation markers Ki67 and PCNA, and balsacone C reduced the expression of Ki67. The antiproliferative effects of phloretin were comparable to those of methotrexate. Phloretin also decreased the levels of various inflammatory cytokines and factors such as IL-17A and TNF-α, while balsacone C and methotrexate had more limited effects. Both phloretin and methotrexate increased the levels of IL-2, suggesting possible polarization of Th17 cells and increased production of T regulatory cells; however, this hypothesis needs to be evaluated with further experiments. The presence of T cells in the cocultures was reduced with the administration of phloretin and methotrexate; CD45 levels were lower. Ultimately, phloretin demonstrated an overall stronger anti-inflammatory activity. This molecule is very promising for psoriasis therapy. Future studies with the administration of phloretin in a 3D psoriatic skin model will be required to investigate its efficacy in reducing epidermal thickness and infiltration of T cells in the skin.

## Figures and Tables

**Table 1:** Median inhibitory concentrations (IC_50_) from the SRB assay and cellular metabolic activity levels from the MTT assay.

Compound	IC_50_ (μM) ^a^	Cellular Metabolic Activity (%) ^b^
Balsacone C (BALS C)	125	61
Phloretin (PHLO)	166	73

^a^ IC_50_ values obtained from the data of three psoriatic lesional keratinocyte donors (N = 3 donors, n = 6 cultures per condition) using the SRB assay. ^b^ Cellular metabolic activity obtained from the data of three psoriatic lesional and two healthy keratinocyte donors (N = 5 donors, n = 6 cultures per condition) treated at the IC_50_ of each molecule, using the MTT assay.

**Table 2:** Information about the donors with plaque psoriasis.

Age	Sex	Region of the Biopsy	Percentage of Body Surface Involved	Treatments Received
36	Female	Back	5% of the body	NA
46	Male	Lower back	NA	NA
49	Male	Back	10% of the body	Methotrexate
65	Female	Back	20% of the body	PUVA therapy, methotrexate, alefacept, and under methotrexate on the day of the biopsy
69	Female	Back	15% of the body	Methotrexate and UVB before/biopsy performed before the new treatment

NA: data not available.

## Data Availability

All relevant data are within this manuscript.

## Supplementary Materials

The following supporting information can be downloaded at: https://www.mdpi.com/article/10.3390/ijms25115639/s1.

## Abbreviations

Healthy control (H CTL), psoriatic control (P CTL), methotrexate (MTX), phloretin (PHLO), balsacone C (BALS C).
