# Supplementary material for: Antiproliferative and Anti-Inflammatory Effects of the Polyphenols Phloretin and Balsacone C in a Coculture of T Cells and Psoriatic Keratinocytes
Source: Int J Mol Sci. 2024 May 22;25(11):5639. doi: 10.3390/ijms25115639 (PMC11171971; doi:10.3390/ijms25115639)
Supplement: Supplementary file 1 [file ijms-25-05639-s001.zip › ijms-2959556-supplementary.pdf]

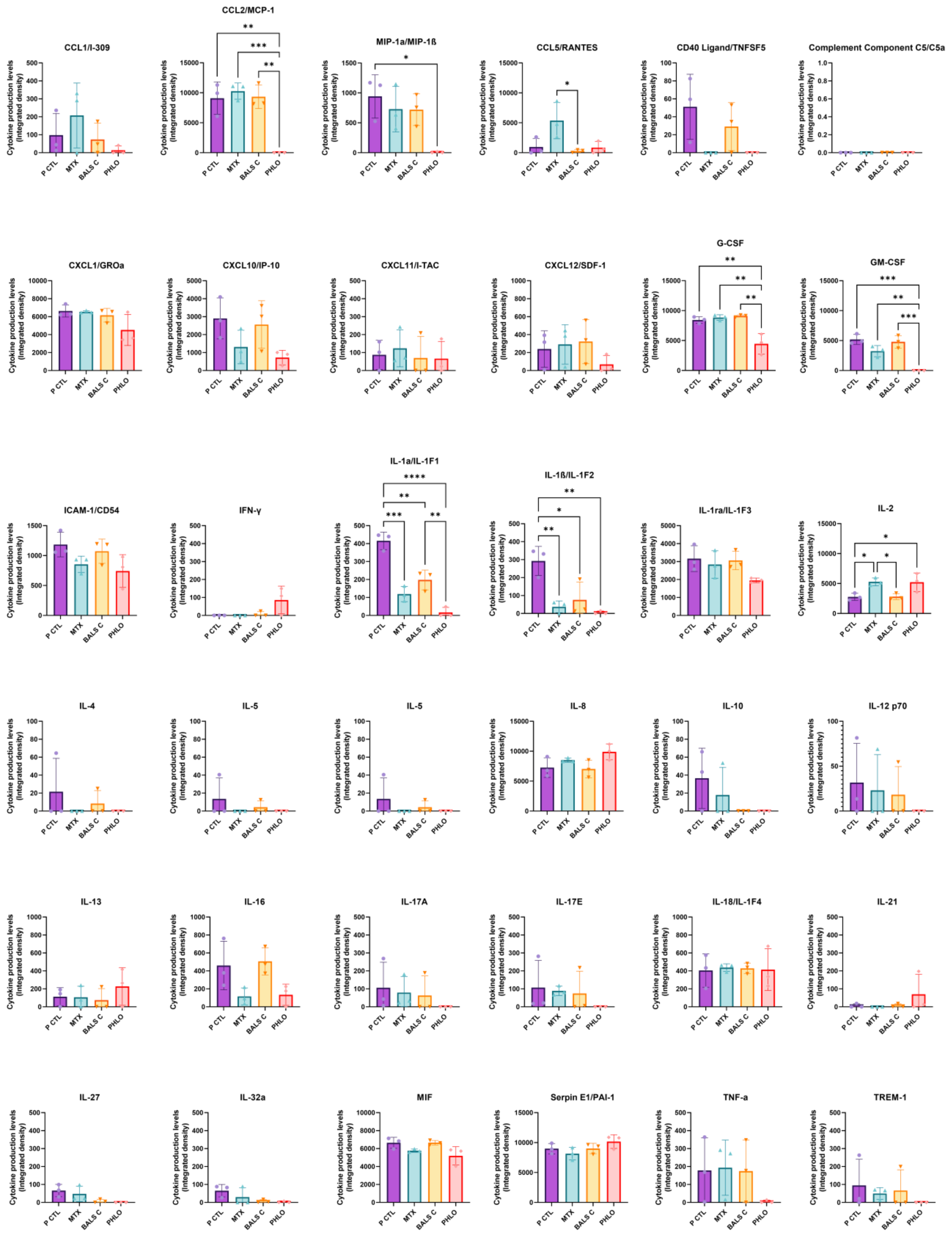

Figure S1. Levels of different inflammatory cytokines in the supernatant of T cell and psoriatic keratinocyte cocultures. The Proteome Profiler Human Cytokine Array kit from R&D Systems was used to detect 36 cytokines in culture supernatants from P CTL, MTX, BALS C and PHLO.

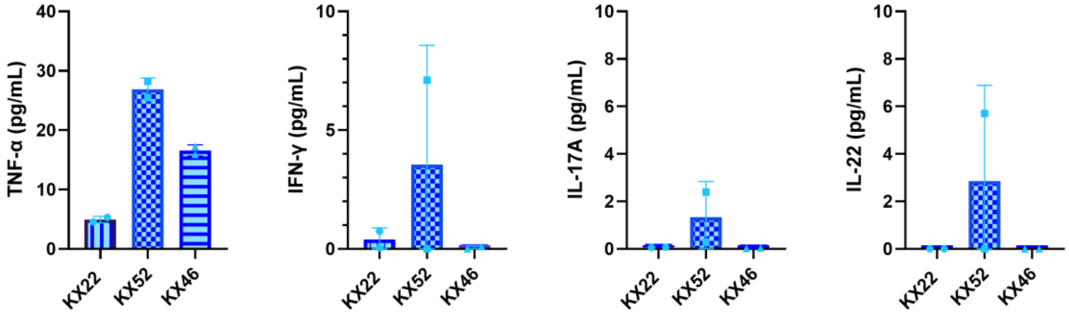

Figure S2. Healthy inter-donor variation noted in ELISA analyses of the supernatant of T cell and psoriatic keratinocyte cocultures.

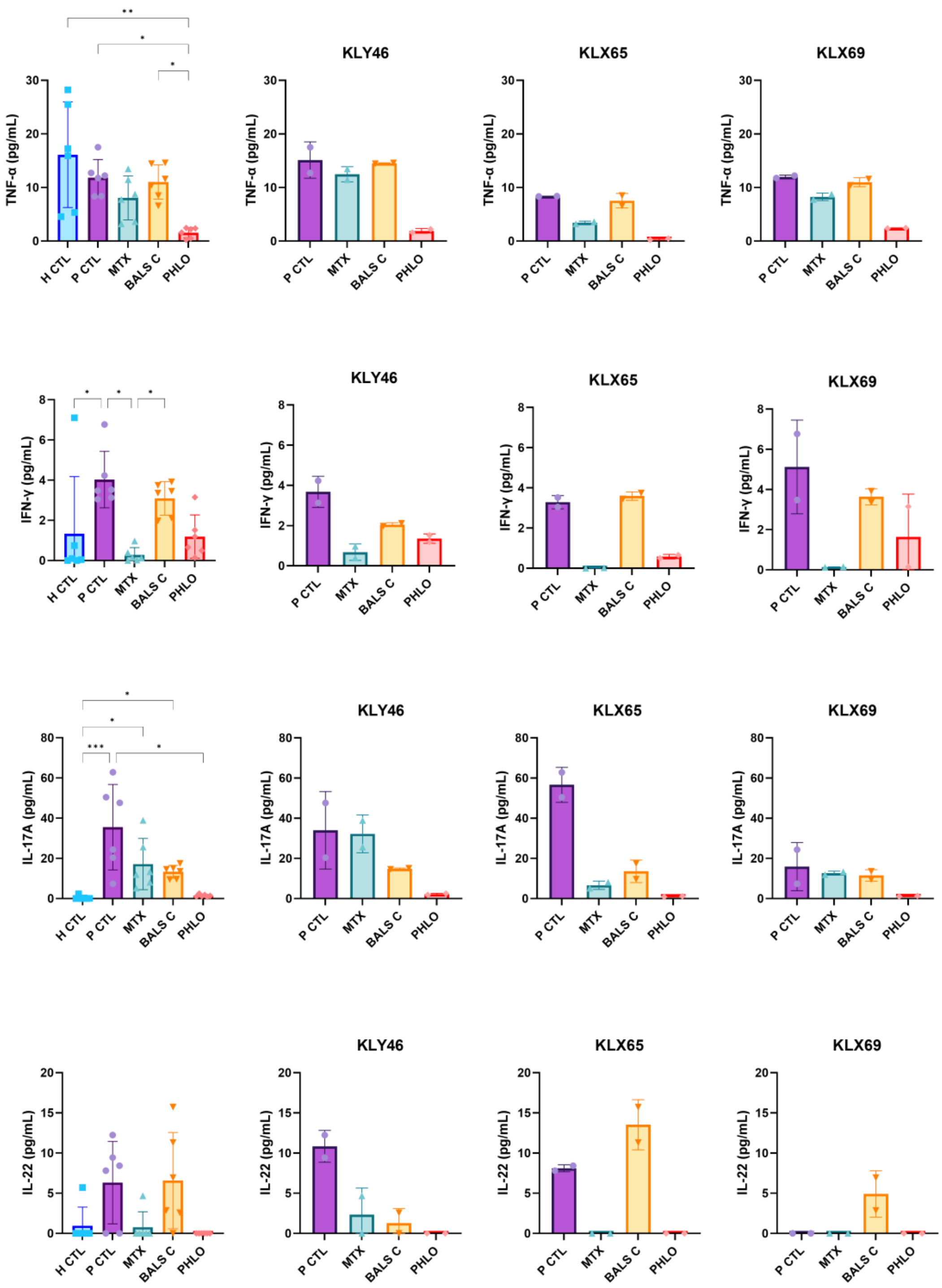

Figure S3. Psoriatic inter-donor variation noted in ELISA analyses of the supernatant of T cell and psoriatic keratinocyte cocultures (with all the different conditions; P CTL, MTX, BALS C, PHLO).
